# Neurosensory component of hand–arm vibration syndrome: a 22-year follow-up study

**DOI:** 10.1093/occmed/kqz029

**Published:** 2019-03-21

**Authors:** L Aarhus, K B Veiersted, K-C Nordby, R Bast-Pettersen

**Affiliations:** 1 Department of Occupational Medicine and Epidemiology, National Institute of Occupational Health, Oslo, Norway; 2 Department of Work Psychology and Physiology, National Institute of Occupational Health, Oslo, Norway

**Keywords:** Hand–arm vibration syndrome, musculoskeletal, neurosensory, pain, Stockholm scale

## Abstract

**Background:**

Knowledge about the long-term course of the neurologic component of hand–arm vibration syndrome (HAVS) is scarce.

**Aims:**

To study the course and prognostic factors of the neurosensory component of HAVS over a period of 22 years.

**Methods:**

Forty male sheet metal workers, with a mean age of 60 (range 45–78) years at follow-up, were examined with a test battery in 1994 and 2017. At baseline, the sample comprised 27 workers with HAVS symptoms and 13 workers without HAVS symptoms. Among the 27 workers, 25 workers reported work-related hand–arm vibration during follow-up (mean 3639 h). In 2017, the mean time since vibration stopped was 8.4 years.

**Results:**

Among the 27 workers with HAVS in 1994, no overall statistically significant change was observed in hand numbness (Stockholm Workshop Scale), shoulder/arm pain (pain scale) or finger pain from 1994 to 2017. However, vibration exposure during follow-up was associated with increased finger pain. Cotinine, carbohydrate-deficient transferrin, glycosylated haemoglobin and folate were not associated with changes in neurosensory symptoms or manual dexterity (Grooved Pegboard) from 1994 to 2017. A diagnosis of HAVS in 1994 did not predict poor hand strength 22 years later. Isolated hand numbness (without white finger attacks) was more common at baseline than at follow-up.

**Conclusions:**

This 22-year follow-up study indicates a tendency towards irreversibility of hand numbness and finger pain in workers with HAVS. Continued vibration exposure seems to predict increased finger pain. Our findings highlight the importance of HAVS prevention.

**Key learning points**

**What is already known:**

- Knowledge about the long-term course of the neurological component of hand–arm vibration syndrome is scarce.
- A few follow-up studies have suggested a tendency towards chronic hand numbness and a degree of improvement in hand pain.
- To our knowledge, no prior study has investigated possible prognostic factors, such as comorbidity and lifestyle factors.

**What this study adds:**

- This 22-year follow-up study of workers with hand–arm vibration syndrome indicates a tendency towards irreversibility in hand numbness and finger pain.
- The workers showed a normal age-related decline in Pegboard score over 22 years of follow-up. A diagnosis of hand–arm vibration syndrome in 1994 was not associated with poor hand strength 22 years later.
- Our study showed no effect of lifestyle factors or comorbidity on the long-term course of neurosensory symptoms in workers with hand–arm vibration syndrome.

**What impact this may have on practice:**

- Our findings highlight the importance of hand–arm vibration syndrome prevention.
- Continued exposure to vibration seems to predict increased finger pain and therefore warrants exposure reduction.
- Numbness occurs with a shorter latency than white finger which and health professionals who follow up of vibration-exposed workers should be mindful of this.

## Introduction

Hand–arm vibration syndrome (HAVS) is associated with peripheral sensorineural, vascular and musculoskeletal symptoms. Knowledge about the long-term course of the neurological component is scarce [1]. A recent systematic review identified three cohort studies of neurosensory HAVS [2], including a study reporting a tendency towards irreversible hand numbness [3], whereas hand pain improved to some extent [3]. To our knowledge, there are no studies of possible prognostic factors, such as comorbidity and lifestyle factors. Previously, we examined the vascular component of HAVS [4]. This study aims to assess the course and prognostic factors of the neurosensory component.

## Methods

In 1994, all employees (*n* = 211) in two workshop units (sheet metal workers performing grinding/welding and machinists) of an engineering and construction company participated in a HAVS examination [4]. We used the 1994 questionnaire to select workers for the 2017 study. Workers who reported previous work-related exposure to hand–arm vibration (HAV), symptoms of hand numbness and/or white finger attacks were classified as the ‘HAVS group’. Those who answered ‘no’ to these three items were classified as the ‘non-HAVS’ group. Participation was voluntary. Written informed consent was obtained. The Regional Ethics Committee, South-East-B approved the study.

We interviewed the workers about work and leisure exposure to hand-transmitted vibration (tool, hours per day, days per year, number of years). Exposure was calculated as hours of occupational vibration exposure during follow-up. Neurosensory symptoms were assessed by the Stockholm Workshop Scale (SWS) [5,6]: 0 = no problems; 1 = periodically recurring numbness/paraesthesia; 2 = commonly recurring numbness/paraesthesia; 3 = constant numbness marked by reduced sensitivity, clumsiness and reduced dexterity. Scores for shoulder/arm pain and finger pain were assessed by the questions ‘Over the past five days, have you felt pain in your shoulder or arm?’ and ‘Over the past five days, have you felt pain in your fingers?’ Response alternatives were no pain/a bit of pain/some pain/quite a lot of pain (score range: 0–3). The carpal tunnel syndrome diagnosis was based on a prior known diagnosis of the condition.

The Grooved Pegboard test (manual dexterity) was used in 1994 and 2017. Other quantitative tests were used in 2017: the finger tapping test (fine motor speed), a hand dynamometer (strength) (all Lafayette Instrument Company) and a hydraulic pinch gauge (strength) (Saehan Corporation). The vibration measurements are described in the ISO 1309-1/2 standard. Blood samples (cotinine, carbohydrate-deficient transferrin (CDT), glycosylated haemoglobin (HbA1c) and folate) were collected during the examination in 2017, as described previously [4].

Wilcoxon signed rank test was used to estimate overall changes from 1994 to 2017 in hand numbness (SWS score), shoulder/arm pain (pain scale), finger pain and manual dexterity. Linear regression was used to assess associations between each prognostic factor (HAV during follow-up, cotinine, CDT, HbA1c and folate) and changes in the outcome variables (the 1994 value subtracted from the 2017 value), with and without age adjustment (IBM SPSS Statistics for Windows, Version 25.0. Armonk, NY: IBM Corp.).

## Results

Of 110 workers who met the inclusion criteria (68/42 with/without HAVS in 1994), two were confirmed dead, and 47 could not be located. Of the 61 invited participants, 21 declined: 11 of 38 (71%) in the HAVS group and 10 of 23 (57%) without HAVS in 1994. Reasons included long travel time or lack of time. Therefore, 40 of 61 (66%) subjects participated in 2017: 27 with HAVS and 13 without HAVS at baseline. Table 1 presents background data and test results for the total sample (*n* = 40). Table 2 presents longitudinal results for the HAVS group (*n* = 27).

**Table 1.** Descriptive data and test results in 2017 for the total sample (*N* = 40)

	HAVS in 1994 (*n* = 27)	No HAVS in 1994 (*n* = 13)	95% CI of the difference
	Mean (SD)	Mean (SD)	
Background data			
Age	60 (10)	61 (11)	
Occupational HAV exposure 1994–2017, in hours	3639 (4685)	606 (2034)	
Vibration-exposed subjects 1994–2017 (*n*)	25	8	
Smokers at last examination (*n*)	4	3	
Pack-years of smoking 1994–2017	4.1 (6.4)	3.0 (5.9)	
Cotinine, µg/L	103 (227)	104 (176)	
CDT, %	0.9 (0.7)	0.8 (0.3)	
HbA1c, %	5.6 (0.7)	5.9 (1.0)	
Folate, nmol/L	12.6 (9.6)	13.6 (7.2)	
Quantitative test results			
Grooved Pegboard, dominant hand	72 (18)	68 (10)	−15.9 to 6.6
Grooved Pegboard, non-dominant hand	80 (21)	75 (16)	−18.6 to 7.9
Finger tapping, dominant hand	50 (10)	51 (5)	−5.3 to 6.8
Finger tapping, non-dominant hand	46 (9)	48 (7)	−3.7 to 7.8
Dynamometer, dominant hand	47 (10)	45 (8)	−8.4 to 4.9
Dynamometer, non-dominant hand	45 (10)	42 (10)	−9.4 to 4.4
Pinch gauge, dominant hand	11.2 (2.3)	11.7 (2.4)	−1.2 to 2.1
Pinch gauge, non-dominant hand	11.4 (2.4)	11.7 (2.1)	−1.3 to 1.9
Vibrometry, standard index, right, 2 Digitus	0.75 (0.12)	0.81 (0.15)	−0.04 to 0.15
<0.8 (%)	17 (63 %)	4 (31 %)	
Vibrometry, standard index, left, 2 Digitus	0.85 (0.12)	0.85 (0.07)	−0.07 to 0.08
<0.8 (%)	9 (33 %)	3 (23 %)	

Pinch gauge, dynamometer and finger tapping scores: a higher score indicates a better performance. Pegboard score: a higher score indicates a worse performance. Missing data: one subject in the group without HAVS in 1994 (*n* = 13) had missing values for all dominant hand test scores.

**Table 2.** Longitudinal results for the workers diagnosed with HAVS in 1994 (*n* = 27)

	1994 study	2017 study
Numbness/white finger attacks/both, *n*	11/4/12	6/2/9
SWS score (SD)	0.7 (0.8)	1.1 (1.1)
Self-reported hand numbness^a^, *n* (%)	18 (67)	15 (56)
Shoulder/arm pain^b^, *n* (%)	14 (52)	13 (48)
Pain level (scale 0–3), mean (SD)	0.9 (1.1)	0.7 (0.8)
Finger pain, *n* (%)	8 (30)	6 (22)
Pain level (scale 0–3), mean (SD)	0.4 (0.8)	0.4 (0.8)
Carpal tunnel syndrome, *n* (%)	5 (19)	4 (15)
Grooved Pegboard, dominant hand, mean (SD)	67 (13)	72 (18)
Grooved Pegboard, non-dominant hand, mean (SD)	72 (13)	80 (21)
Two-point discrimination >2 mm	5 (15)	4 (19)

Pegboard score: A higher score indicates a worse performance.

^a^During the last period of time, have you felt hand numbness? yes/no.

^b^‘Over the past five days, have you felt pain in your shoulder or arm?’ and ‘Over the past five days, have you felt pain in your fingers?’ no pain = no; a bit of pain/some pain/quite a lot of pain = yes.

The 27 workers exhibited an overall reduced performance in Pegboard scores from 1994 to 2017: 5 s for the dominant hand (*Z* = −2.4, *P* < 0.05); 8 s for the non-dominant hand (*Z* = −2.5, *P* < 0.05). No statistically significant changes in hand numbness, arm pain or finger pain scores were found.

Age-adjusted regression analyses showed that 1000 h of HAV exposure during follow-up predicted an increase of 0.12 (95% confidence interval (CI) 0.06–0.17) in finger pain score (pain scale score 0–3) from 1994 to 2017. Cotinine predicted a larger deterioration in Pegboard scores from 1994 to 2017: dominant/non-dominant hand: unstandardized *B* 0.016 (95% CI 0.002–0.030)/0.030 (0.002–0.058).

## Discussion

Workers diagnosed with HAVS in 1994 showed no statistically significant change in arm or finger pain over 22 years. However, vibration exposure during follow-up predicted increased pain at follow-up. A previous follow-up study of travertine workers showed that hand pain was partially reversible [3]. Workers with HAVS often report pain in the upper extremity [7], hands and fingers [8]. Pain seems to be an important aspect of HAVS and should probably be given increased attention in the symptom-driven diagnosis and staging of neurosensory HAVS.

Our finding of no significant changes in hand numbness over 22 years is consistent with the above-mentioned study [3]. Another study of workers in whom exposure to chainsaw vibration was reduced during follow-up showed some improvement in hand numbness and complaints of hand muscle weakness [9]. Regarding manual dexterity, the present Pegboard score declined during 22 years of follow-up in agreement with published reference values when allowing for aging [10].

To our knowledge, no study has examined the effect of lifestyle factors and comorbidities on the course of neurosensory HAVS; in this study, the effect of smoking (cotinine), alcohol consumption (CDT), glucose metabolism (HbA1c) and folate deficiency (folate) (all established risk factors of polyneuropathy) showed no influence.

One weakness of this study is that the negative findings may be due to the small number of subjects (type 2 error). The longitudinal design, however, has more statistical power than a cross-sectional design as due to the longer observation time; it uses repeated observations at individual levels. It also reduces selection bias and unmeasured confounding, so negative findings are easier to interpret. Intra-observer bias was reduced by using the same interviewers and standardized questions, and following a structured protocol. HAV exposure however was based on self-reports, which may introduce recall bias.

Finally, our descriptive data showed that isolated hand numbness without white finger attacks was more common in 1994 than in 2017, consistent with Nilsson *et al.*’s report that neurosensory injury occurs with a shorter latency than Raynaud’s phenomenon [2].
